# Benefício de Prestação Continuada e envelhecimento no Brasil: significados sociais da proteção de renda e fricções institucionais na implementação

**DOI:** 10.1590/0102-311XPT033126

**Published:** 2026-10-30

**Authors:** Victor Matheus Queiroz de Paz, Alexander Itria

**Affiliations:** 1 Universidade Federal de São Carlos, São Carlos, Brasil.

**Keywords:** Pessoas Idosas, Proteção Social, Equidade, Benefícios, Elderly, Social Protection, Equity, Fringe Benefits, Personas Mayores, Protección Social, Equidad, Beneficios

## Abstract

Este estudo analisa em que medida o Benefício de Prestação Continuada (BPC) promove segurança econômica e equidade social para pessoas idosas vulneráveis no Brasil, considerando desigualdades estruturais, judicialização e restrições fiscais e administrativas. É uma pesquisa qualitativa, exploratória e descritiva, baseada em revisão integrativa da literatura e análise de dados secundários de fontes oficiais como o Instituto Brasileiro de Geografia e Estatística, o Instituto de Pesquisa Econômica Aplicada, o Cadastro Único, Secretaria de Avaliação, Gestão da Informação e Cadastro Único e Tribunal de Contas da União, além de legislação e decisões judiciais do Supremo Tribunal Federal e do Superior Tribunal de Justiça, com recorte temporal de 2010 a 2025. Os achados indicam que o BPC exerce uma função consistente de proteção não contributiva, reduzindo a exposição imediata à privação material, especialmente entre idosos com trajetórias laborais informais e baixa densidade contributiva. No entanto, a contribuição para a equidade não é automática, pois persistem barreiras de acesso e permanência. A judicialização emerge como um componente estrutural: embora minoritárias, as concessões judiciais expandem-se de forma contínua e relativamente acelerada, configurando via recorrente de contestação de indeferimentos e disputa sobre critérios e provas. Além disso, oscilações no tempo médio de concessão evidenciam fragilidade institucional, com melhora em 2024 e deterioração em 2025, sugerindo instabilidade da capacidade administrativa. Conclui-se que o BPC fortalece a segurança econômica de pessoas idosas vulneráveis, mas sua capacidade de promover equidade depende de arranjos de governança, estabilidade da capacidade estatal e redução de fricções institucionais, incluindo aquelas associadas à judicialização e às filas de análise.

## Abstract (en)

This study analyzes to what extent the Brazilian Continuous Cash Benefit (BPC, acronym in Portuguese) promotes economic security and social equity for vulnerable older adults in Brazil, considering structural inequalities, judicialization, and fiscal and administrative constraints. This is a qualitative, exploratory, descriptive study, based on an integrative literature review and analysis of secondary data from official sources such as the Brazilian Institute of Geography and Statistics, the Institute for Applied Economic Research, the Unified Registry, the Secretariat for Evaluation, Information Management and the Unified Registry, and The Federal Court of Accounts, as well as legislation and judicial decisions of the Supreme Federal Court and the Superior Court of Justice, covering the period from 2010 to 2025. The findings indicate that the BPC plays a consistent non-contributory income protection role, reducing immediate exposure to material deprivation, especially among older adults with informal work trajectories and low contributory density. However, the contribution to equity is not automatic, as barriers to access and benefit retention persist. Judicialization emerges as a structural component: although they represent a minority of cases, judicially granted benefits expand continuously and at a relatively accelerated pace, constituting a recurrent avenue for challenging denials and for disputes over criteria and evidence. Moreover, fluctuations in the average processing time for granting benefits reveal institutional fragility, with an improvement in 2024 and a deterioration in 2025, suggesting instability in administrative capacity. It is concluded that the BPC strengthens the economic security of vulnerable older adults, but its capacity to promote equity depends on governance arrangements, stability of state capacity, and the reduction of institutional frictions, including those associated with judicialization and backlogs in application processing.

## Resumen

Este estudio analiza en qué medida el Beneficio de Prestación Continuada (BPC) promueve la seguridad económica y la equidad social a las personas mayores vulnerables en Brasil al tener en cuenta las desigualdades estructurales, la judicialización y las restricciones fiscales y administrativas. Se trata de una investigación cualitativa, exploratoria y descriptiva, basada en una revisión integradora de la literatura y análisis de datos secundarios de fuentes oficiales como el Instituto Brasileño de Geografía y Estadística, el Instituto de Investigación Económica Aplicada, el Registro Único, la Secretaría de Evaluación, Gestión de la Información y Registro Único, y el Tribunal Federal de Cuentas, además de normativa y decisiones judiciales del Supremo Tribunal Federal y del Superior Tribunal de Justicia, con un marco temporal de 2010 a 2025. Los hallazgos indican que BPC tiene una función consistente de protección no contributiva, lo cual reduce la exposición inmediata a la privación material, especialmente entre las personas mayores con trayectorias laborales informales y baja densidad de contribución. Sin embargo, la contribución a la equidad no es automática, ya que persisten las barreras de acceso y permanencia. La judicialización surge como un componente estructural: aunque minoritarias, las concesiones judiciales se expanden de manera continua y relativamente rápida, lo cual configura una forma recurrente de impugnar denegaciones y disputas sobre criterios y pruebas. Además, las variaciones en el tiempo promedio de concesión muestran fragilidad institucional, con mejora en 2024 y deterioro en 2025, lo que sugiere inestabilidad de la capacidad administrativa. Se concluye que BPC fortalece la seguridad económica de las personas mayores vulnerables, pero su capacidad para promover la equidad depende de los mecanismos de gobernanza, de la estabilidad de la capacidad estatal y de la reducción de las fricciones institucionales, incluidas las asociadas con la judicialización y las listas de espera de análisis.

## Introdução

O envelhecimento populacional constitui uma das transformações demográficas mais expressivas do século XXI e impõe exigências estruturais às políticas de proteção social em contextos de marcada desigualdade socioeconômica, como o Brasil ^1^. A proporção de pessoas com 60 anos ou mais já ultrapassa 15% da população nacional e projeta um crescimento acelerado nas próximas décadas, ampliando a demanda por mecanismos institucionais que assegurem renda, autonomia e dignidade na velhice ^2^.

O Benefício de Prestação Continuada (BPC) ocupa posição central nesse arranjo. Instituído pelo artigo 203 da *Constituição Federal* de 1988 ^3^ e regulamentado pela Lei Orgânica da Assistência Social (LOAS - *Lei nº 8.742/1993*) ^4^, o BPC é uma transferência de renda não contributiva, de natureza assistencial, gerida pelo Instituto Nacional do Seguro Social (INSS) no âmbito da proteção social básica do Sistema Único de Assistência Social (SUAS) ^3^.

Para a população idosa, o benefício é assegurado a pessoas com 65 anos ou mais que comprovem não possuir meios de prover a própria manutenção ou de tê-la provida pela família, adotando como critério de elegibilidade a renda familiar *per capita* igual ou inferior a um quarto do salário mínimo vigente. O benefício corresponde a um salário mínimo mensal e não exige contribuição prévia ao sistema previdenciário, distinguindo-se das aposentadorias do Regime Geral de Previdência Social ^3^.

Tal configuração o torna pilar da proteção não contributiva voltada a indivíduos cuja trajetória laboral, frequentemente marcada por informalidade, descontinuidade e baixa densidade contributiva, não garante acesso a benefícios previdenciários regulares ^5^. A segurança econômica, conceito central neste estudo, é compreendida como a capacidade de manter nível adequado de renda e de acesso a recursos ao longo do tempo, preservando a autonomia e evitando a privação material grave.

Na perspectiva de Amartya Sen ^6^, a segurança econômica é condição para a expansão das liberdades substantivas, as oportunidades reais que os indivíduos têm de viver a vida que valorizam. Na velhice, sua ausência compromete o acesso à alimentação, a medicamentos, à moradia e cuidados, com impactos diretos sobre a saúde e o bem-estar. A equidade social, por sua vez, é aqui entendida com base no referencial rawlsiano como a distribuição justa de bens e oportunidades, em que desigualdades são admissíveis somente quando beneficiam os menos favorecidos, o denominado “princípio da diferença” ^7^.

Aplicado ao BPC, esse referencial implica questionar não apenas se o benefício existe como dispositivo legal, mas se ele efetivamente alcança, de forma equânime, os que mais dele necessitam. Essa distinção é fundamental porque, em políticas focalizadas, a equidade não é atributo automático: depende de como o direito é operacionalizado no cotidiano das instituições, de como os critérios são interpretados e de como diferentes grupos conseguem, ou não, atravessar as etapas burocráticas necessárias para acessar o benefício ^8^.

O “custo burocrático” do acesso pode recair de forma desproporcional sobre quem tem menor escolaridade, menor capital informacional, maior limitação funcional ou menor rede de apoio, perfil que caracteriza uma parcela relevante das pessoas idosas em situação de pobreza. Assim, o benefício pode coexistir com a exclusão administrativa parcial: o programa “existe”, mas a porta de entrada não é igualmente transponível por todos ^9^.

Um elemento central desse debate é a judicialização. A presença crescente de concessões judiciais indica que controvérsias sobre critérios e padrões de prova são parte constitutiva do funcionamento do benefício. Em termos analíticos, a judicialização não é apenas um “desvio” do fluxo administrativo: ela pode ser interpretada como um mecanismo pelo qual o sistema jurídico participa da efetivação do direito quando há disputas sobre interpretação normativa, definição de vulnerabilidade ou avaliação de evidências ^10^.

Esse fenômeno tem implicações distributivas importantes: quando a obtenção do benefício depende de litígio, a efetivação do direito pode tornar-se mais provável para indivíduos com maior capacidade de mobilização jurídica, acesso à informação e suporte institucional, reproduzindo desigualdades na trajetória de acesso ^11^. Além disso, o tempo de processamento dos requerimentos constitui um indicador substantivo da qualidade de implementação.

Em políticas de renda mínima, o atraso não é apenas variável administrativa: ele é componente social da efetividade do direito. Filas prolongadas implicam que a renda chegue tarde, agravando a insegurança econômica e ampliando o risco de estratégias de sobrevivência nocivas, como endividamento financeiro, restrição alimentar, desistência do processo ^12^.

Diante disso, este artigo analisa em que medida o BPC promove segurança econômica e equidade social para pessoas idosas vulneráveis no Brasil, enfatizando como desigualdades estruturais, judicialização e fricções institucionais modulam o acesso e a efetividade do benefício no período de 2019 a 2025. Ao articular o perfil dos beneficiários, as vias de concessão e o desempenho operacional, busca-se oferecer uma interpretação centrada na implementação, útil para a agenda de saúde coletiva, na medida em que a proteção de renda integra os determinantes sociais da saúde.

## Métodos

### Delineamento e justificativa metodológica

É um estudo exploratório e descritivo, baseado em revisão integrativa da literatura articulada à análise de dados secundários públicos e documentos institucionais. Os dados analisados são predominantemente de natureza quantitativa, como séries administrativas, registros de beneficiários e estatísticas de concessões ^13^. A revisão integrativa é adequada a este objeto por permitir combinar evidências de múltiplas abordagens e fontes acadêmicas, técnicas e administrativas, favorecendo síntese abrangente sobre um programa complexo ^14^.

### Justificativa do recorte temporal

O período de análise compreende janeiro de 2019 a dezembro de 2025. Janeiro de 2019 marcou o início do Governo Federal que adotou a postura de contenção do BPC, com tentativas de redução do valor e elevação da idade mínima de acesso, criando um ponto de inflexão relevante na série histórica de concessões. O recorte é estendido até dezembro de 2025, capturando os efeitos do Novo Arcabouço Fiscal e das revisões cadastrais realizadas entre 2023 e 2025. O intervalo de seis anos permite examinar ciclos de expansão, contração e oscilação operacional centrais à compreensão das fricções institucionais investigadas.

### Etapas da revisão integrativa

A revisão foi conduzida em conformidade com o protocolo de cinco etapas preconizado por Souza et al. ^14^:

(1) Identificação do problema. A questão norteadora foi: em que medida o BPC promove segurança econômica e equidade social para as pessoas idosas vulneráveis no Brasil, considerando-se as desigualdades estruturais, a judicialização e as fricções administrativas no período 2019-2025?

(2) Busca de evidências. Foram realizadas buscas nas bases SciELO, Periódicos CAPES e Google Scholar, com os descritores: “Benefício de Prestação Continuada”, “BPC idoso”, “judicialização”, “transferência de renda”, “envelhecimento”, “proteção social”, “Brasil”. Buscas complementares foram realizadas nos repositórios do Instituto de Pesquisa Econômica Aplicada (IPEA), do Ministério do Desenvolvimento e Assistência Social, Família e Combate à Fome (MDS) e do Instituto Brasileiro de Geografia e Estatística (IBGE). O recorte bibliográfico contemplou publicações entre 2010 e 2025, excetuando obras de referência teórica e marcos normativos históricos. Foram considerados materiais em português, inglês e espanhol.

(3) Avaliação dos dados. Critérios de inclusão: publicações entre 2010 e 2025; materiais que abordam diretamente o BPC, políticas de transferência de renda, envelhecimento ou referenciais teóricos relacionados; fontes com rigor metodológico identificável. Foram excluídos os textos opinativos sem fundamentação técnica e publicações sem caráter de referência estruturante anteriores a 2010.

(4) Análise e síntese. As evidências foram organizadas em três eixos temáticos: perfil sociodemográfico e marcadores de desigualdade; acesso administrativo e judicialização; e desempenho operacional. A triangulação entre fontes foi usada para evitar leituras simplistas, reconhecendo que séries administrativas descrevem padrões e variações, mas demandam interpretação contextual.

(5) Apresentação dos resultados. Os achados foram organizados nos três eixos mencionados, articulando indicadores administrativos ao debate acadêmico e normativo.

### Fontes de dados e operacionalização das medidas

(a) Perfil sociodemográfico dos beneficiários idosos: dados extraídos da Secretaria de Avaliação, Gestão da Informação e Cadastro Único (SAGICAD/MDS) ^15^, com referência temporal de dezembro de cada ano do período 2019-2025. Para qualificar a interpretação da distribuição etária e por sexo, os dados absolutos foram contextualizados com base na estrutura demográfica da pirâmide etária brasileira obtida da *Pesquisa Nacional por Amostra de Domícilios - Contínua* (PNAD Contínua) do IBGE ^16^. Reconhece-se que comparações mais precisas de cobertura demandariam o cálculo da proporção de beneficiários em relação à população total em cada faixa etária e sexo (número de beneficiários na faixa/sexo ÷ população na faixa/sexo × 10ⁿ), análise indicada como aperfeiçoamento para estudos futuros.

(b) Evolução do estoque de benefícios: série anual de beneficiários idosos do BPC extraída da SAGICAD/MDS, com dados de dezembro de cada ano entre 2019 e 2025.

(c) Concessões por via administrativa e judicial: dados mensais extraídos da plataforma VIS DATA do MDS ^17^. A análise descreve a evolução em números absolutos e tendências de crescimento relativo. O emprego de medidas relativas (beneficiários/população maior de 65 anos × 10ⁿ) e técnicas de regressão linear segmentada (*joinpoint*) são indicados para estudos futuros.

(d) Tempo médio de concessão: série mensal expressa em dias, extraída da plataforma Monitora MDS ^12^, com início em janeiro de 2019. Essa série foi empregada como indicador de capacidade operacional e de fricção institucional. A unidade “dias” é explicitamente adotada em toda a análise e nas figuras correspondentes.

(e) Documentos normativos e institucionais: *Constituição Federal* (Art. 203), LOAS (*Lei nº 8.742/1993*), *Decreto nº 6.214/2007* ^18^ e alterações subsequentes, além de relatórios técnicos do Tribunal de Contas da União (TCU) e do IPEA.

### Considerações éticas

O estudo utiliza exclusivamente dados secundários públicos, disponíveis em plataformas de acesso aberto, sem identificação individual e sem interação com participantes, estando dispensado de submissão a comitê de ética em pesquisa, nos termos da *Resolução nº 510/2016* do Conselho Nacional de Saúde. A análise foi conduzida com compromisso de transparência e rastreabilidade das informações.

## Resultados

### Perfil dos beneficiários idosos e marcadores de desigualdade

A evolução do estoque de beneficiários idosos do BPC indica um crescimento expressivo ao longo do período. Em dezembro de 2019, havia 2.046.710 beneficiários; ao final de 2024, esse número alcançou 2.723.393, crescimento de aproximadamente 33,1%. As variações anuais foram mais contidas entre 2019 e 2021 (em torno de 2,6% e 2,8% ao ano); a partir de 2022, observou-se aceleração mais intensa, com aumentos de 8,6% em 2022 e 10,2% em 2023. Em dezembro de 2025, registrou-se retração para 2.661.161 beneficiários (-2,3% em relação a 2024), ainda que o patamar permanecesse aproximadamente 30% acima do registrado em 2019.

A distribuição etária (Tabela 1) revela concentração entre 65 e 74 anos, faixa que reúne 54,43% dos beneficiários. A partir dos 75 anos, observa-se uma redução progressiva: 75-79 anos correspondem a 22,06%; 80-84 anos, a 14,25%; 85-89 anos, a 6,51%; as faixas acima de 90 anos totalizam 2,7%.

**Tabela 1:** Distribuição dos beneficiários idosos do Benefício de Prestação Continuada (BPC) por faixa etária. Brasil, dezembro de 2024.

Faixa etária (anos)	Participação relativa (%)
65-74	54,43
75-79	22,06
80-84	14,25
85-89	6,51
90 ou mais	2,70

Fonte: elaboração própria com base em Secretaria de Avaliação, Gestão da Informação e Cadastro Único do Ministério do Desenvolvimento e Assistência Social, Família e Combate à Fome (SAGICAD/MDS) ^15^.

Embora a pirâmide etária brasileira apresente afunilamento natural nas idades mais avançadas, com maior mortalidade e menor contingente populacional nessas faixas, a queda proporcional do volume de beneficiários sugere que fatores adicionais podem estar em operação. Uma análise de cobertura proporcional (razão beneficiários/população por faixa etária) seria necessária para distinguir o efeito demográfico de possíveis barreiras de acesso que são intensificadas com o avanço da idade, sendo recomendada para estudos futuros.

A distribuição por gênero (Tabela 2) é particularmente informativa. As mulheres representam a maioria em todo o período: em 2019, 59,5% das beneficiárias eram do sexo feminino; em 2025, essa proporção chegou a 60,3%. Em termos absolutos, a diferença cresceu de aproximadamente 390 mil em 2019 para cerca de 549 mil em 2025. Na retração de 2025, a queda foi mais intensa entre os homens (-2,9%) do que entre as mulheres (-1,8%).

**Tabela 2:** Distribuição dos beneficiários idosos do Benefício de Prestação Continuada (BPC) por sexo. Brasil, 2019-2025.

Ano	Total de beneficiários	Mulheres (%)	Homens (%)	Diferença absoluta (mulheres/homens)
2019	2.046.710	59,5	40,5	~390.000
2025	2.661.161	60,3	39,7	~549.000

Fonte: elaboração própria com base em Secretaria de Avaliação, Gestão da Informação e Cadastro Único do Ministério do Desenvolvimento e Assistência Social, Família e Combate à Fome (SAGICAD/MDS) ^15^.

Nota: em 2025, a queda foi de -2,9% entre homens e -1,8% entre mulheres.

### Acesso administrativo e judicialização do BPC para pessoas idosas

A análise das concessões mensais evidencia que a via administrativa permanece como a principal porta de entrada do benefício, conforme esperado pelo desenho institucional do BPC (Figura 1). Contudo, a via judicial apresenta um crescimento persistente ao longo da série (Figura 2), indicando que a judicialização não é episódica, mas parte de um padrão estrutural de contestação e revisão de decisões administrativas. A inflexão mais pronunciada ocorreu a partir de meados de 2022, período que coincide com o crescimento de processos revertidos no âmbito de Tribunais Regionais Federais, além de alterações normativas sobre a possibilidade de dois benefícios por família ^10^.

**Figura 1:**
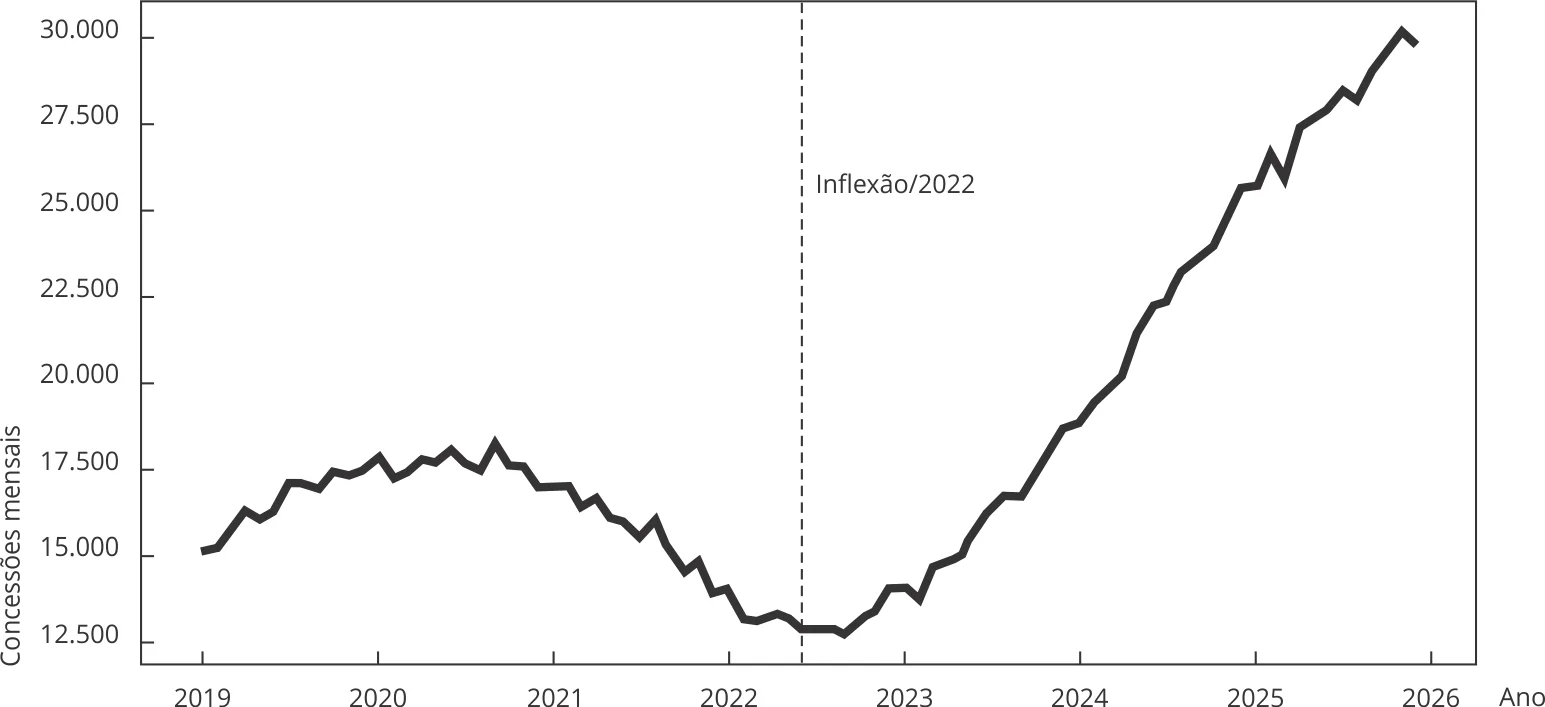
Evolução mensal do número de beneficiários do Benefício de Prestação Continuada (BPC) oriundos de concessão administrativa para pessoas idosas no Brasil, no período de janeiro de 2019 a dezembro de 2025.

Fonte: VIS DATA (Ministério do Desenvolvimento e Assistência Social, Família e Combate à Fome ^17^).

**Figura 2:**
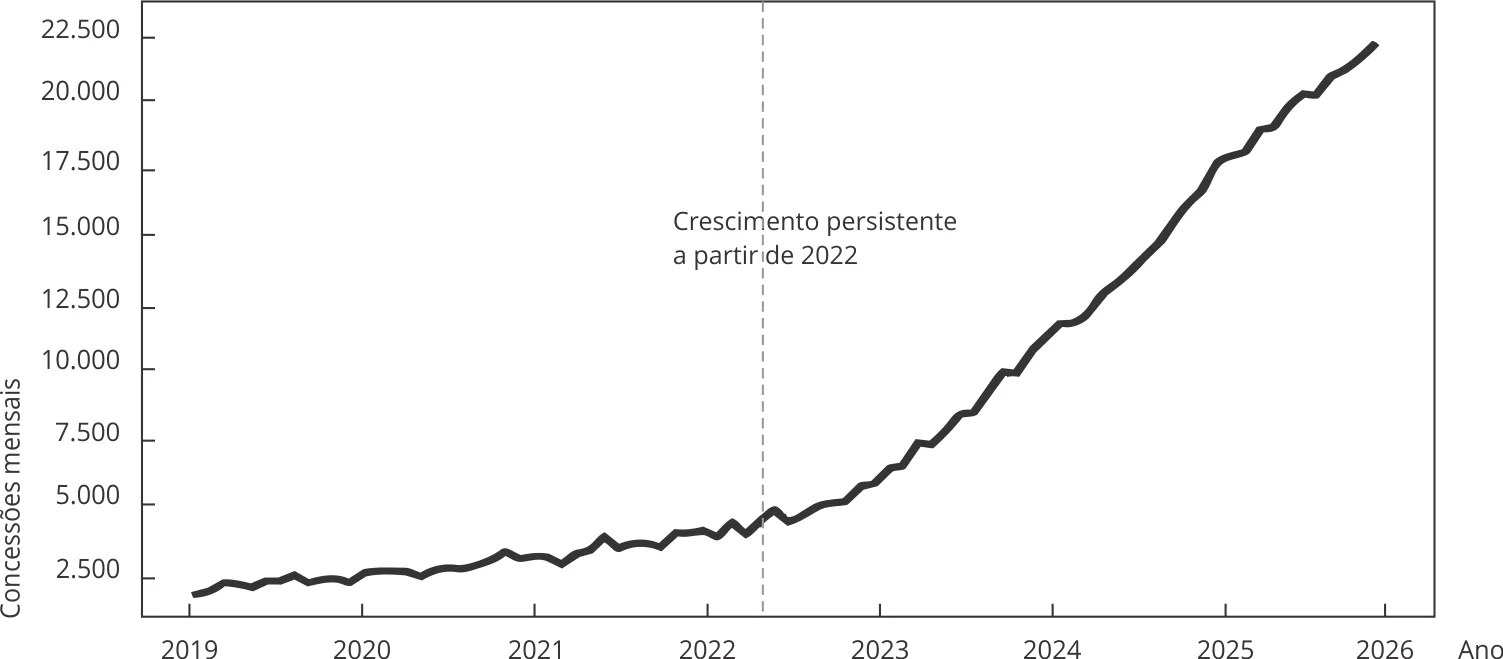
Evolução mensal do número de beneficiários do Benefício de Prestação Continuada (BPC) oriundos de concessão judicial para pessoas idosas no Brasil, no período de janeiro de 2019 a dezembro de 2025.

Fonte: VIS DATA (Ministério do Desenvolvimento e Assistência Social, Família e Combate à Fome ^17^).

### Tempo médio de concessão e fricções institucionais

O tempo médio de concessão, expresso em dias, constitui um dos indicadores mais expressivos para avaliar as fricções institucionais na implementação do BPC (Figura 3).

**Figura 3:**
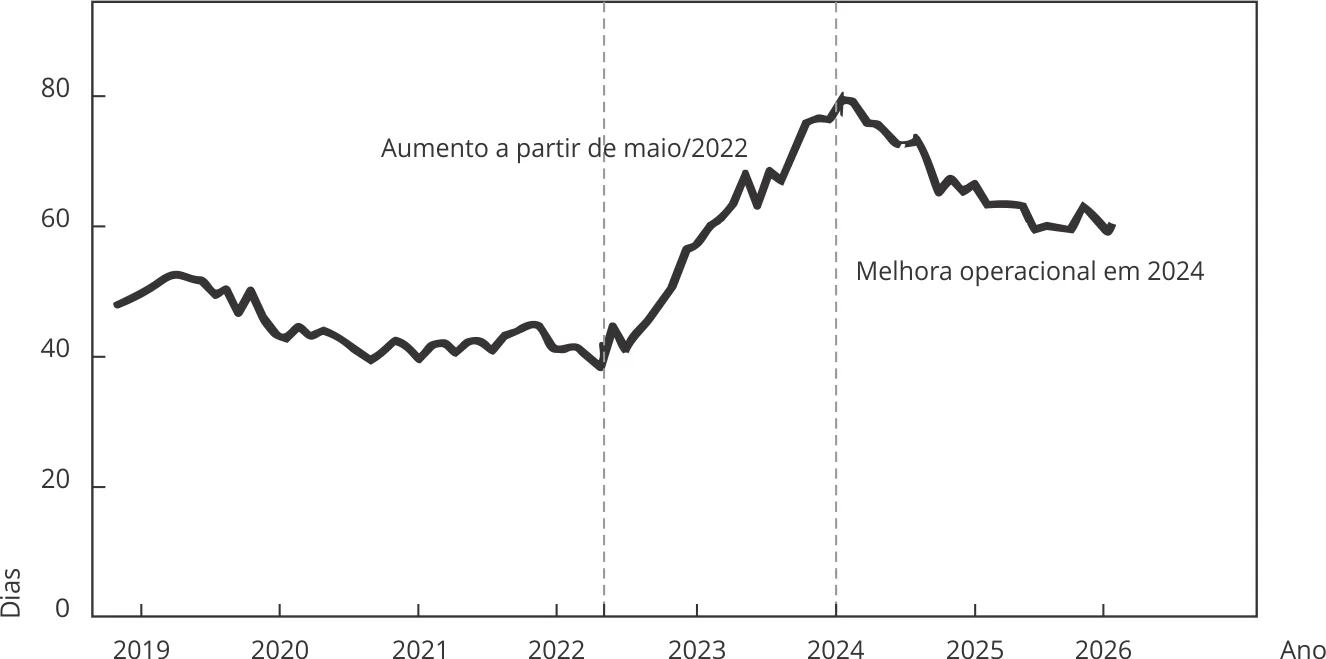
Tempo médio de concessão do Benefício de Prestação Continuada (BPC) para pessoas idosas no Brasil (em dias), no período de janeiro de 2019 a dezembro de 2025.

Fonte: Monitora MDS (Ministério do Desenvolvimento e Assistência Social, Família e Combate à Fome ^12^).

A série revela variações relevantes. A partir de maio de 2022, observou-se um aumento do tempo médio em dias, com oscilações nos períodos subsequentes. Momentos de melhora em 2024 sugerem que reduções no tempo de espera são possíveis por reorganização de fluxos, digitalização ou melhorias pontuais de gestão. No entanto, a deterioração subsequente indica que tais ganhos operacionais podem não ser sustentáveis sem reforços estruturais de capacidade institucional.

## Discussão

Os resultados reafirmam o BPC como um mecanismo decisivo de proteção de renda na velhice, com o potencial de reduzir a vulnerabilidade econômica em um contexto marcado por desigualdades estruturais e insuficiência de cobertura contributiva para parcelas expressivas da população. No entanto, os achados sustentam que a equidade do benefício é mediada por fricções institucionais: o direito formal pode ser restringido, na prática, por barreiras burocráticas, por instabilidades de processamento e por padrões de prova que nem sempre capturam adequadamente as vulnerabilidades complexas ^19^. A consequência é a possibilidade de seletividade prática, um filtro institucional que altera quem acessa, por quais vias e em que tempo.

A concentração de beneficiários na faixa de 65 a 74 anos pode ser interpretada, em parte, como reflexo da estrutura demográfica brasileira, com maior contingente populacional nas idades menos avançadas da velhice. Contudo, uma análise de cobertura proporcional seria necessária para separar o efeito demográfico do efeito de barreiras de acesso que são intensificadas com a idade.

A hipótese de que pessoas muito idosas enfrentam obstáculos adicionais, mobilidade reduzida, maior dependência de terceiros, dificuldade de acesso a canais digitais, obstáculos na atualização do Cadastro Único, é coerente com a literatura e com evidências de que a demanda por cuidados tende a ser intensificada exatamente quando o acesso ao benefício parece declinar ^20^. Em termos de saúde coletiva, essa assimetria é particularmente preocupante: a insuficiência de renda em idades avançadas pode intensificar agravos, reduzir a adesão a cuidados e comprometer a qualidade de vida em um segmento especialmente vulnerável ^21^.

A predominância feminina entre os beneficiários idosos exige leitura que articule estrutura demográfica e desigualdade social. Do ponto de vista demográfico, as mulheres apresentam maior expectativa de vida ao nascer e em idades específicas: dados do IBGE indicam que, ao nascer, a diferença é de aproximadamente sete anos (79,4 *versus* 72,8 anos); aos 65 anos, a expectativa de vida adicional das mulheres supera a dos homens em cerca de três a quatro anos ^16^.

Esse diferencial faz com que a população feminina seja estruturalmente majoritária nas idades de elegibilidade ao BPC, o que explica parte do maior volume de beneficiárias em termos absolutos. No entanto, o crescimento da diferença absoluta ao longo do período e a menor retração feminina observada em 2025 indicam que fatores além da sobrevivência diferencial estão em operação, em particular as desigualdades acumuladas de gênero nas trajetórias laborais ^22^.

Nesse sentido, a presença majoritária de mulheres no BPC pode ser interpretada como evidência de que a proteção assistencial cumpre uma função compensatória parcial diante de desigualdades históricas de gênero ^23^. Ao mesmo tempo, se barreiras burocráticas forem mais intensas entre as mulheres idosas em pobreza, por maior fragilidade de redes de apoio ou dificuldade de mobilidade, a equidade do benefício pode ser comprometida exatamente no grupo mais dependente da política ^24^.

A judicialização emerge como um componente estrutural do ecossistema de implementação ^10^. Seu crescimento persistente indica que a arena judicial participa da efetivação do direito e funciona como mecanismo de revisão de decisões e de disputa interpretativa sobre elegibilidade. Esse fenômeno tem dupla face: de um lado, pode corrigir injustiças e ampliar o acesso em casos concretos, especialmente quando indeferimentos administrativos são baseados em interpretações restritivas da vulnerabilidade; de outro, produz desigualdade na efetivação, visto que acessar a via judicial demanda recursos institucionais e informacionais que não são igualmente distribuídos ^11^.

Assim, a judicialização pode tornar a garantia do direito mais provável para quem tem melhor acesso à mediação jurídica, criando assimetria que contradiz o objetivo redistributivo do programa ^10^ ^,^ ^11^. A persistência do componente judicial sugere que as controvérsias sobre elegibilidade não são resolvidas com ajustes marginais de procedimento: se as concessões judiciais crescem de forma sustentada, há indício de que o modo como a vulnerabilidade é reconhecida administrativamente pode não capturar adequadamente situações de pobreza complexa e multidimensional, o que, no contexto da saúde coletiva, tem relevância direta sobre os determinantes sociais da saúde e sobre as iniquidades no acesso a recursos básicos para a vida.

O tempo médio de concessão em dias amplia essa leitura ao revelar instabilidade de capacidade institucional. A proteção de renda, para ser efetiva, precisa ser previsível e chegar em tempo socialmente oportuno ^20^. Oscilações de desempenho com melhora em determinados momentos e deterioração subsequente sugerem que mudanças operacionais podem ser contingentes e vulneráveis a restrições de pessoal, reorganizações institucionais e pressões de demanda ^25^.

Do ponto de vista distributivo, a instabilidade afeta mais intensamente quem dispõe de menos alternativas: para grupos em pobreza, a demora implica prolongar a privação e a insegurança econômica, com impactos sobre saúde, alimentação e possibilidade de adesão a cuidados ^21^. A articulação entre judicialização e tempo de concessão sugere um circuito potencial: fricções administrativas e atraso podem elevar a demanda judicial, que por sua vez retroalimenta os custos de gestão e as disputas sobre critérios, intensificando a complexidade do ecossistema de implementação.

Nessa perspectiva, o debate sobre o BPC deve ser realocado no centro da agenda de saúde coletiva não apenas como tema de assistência social, mas como um componente estrutural dos determinantes sociais da saúde ^26^. A garantia de renda mínima funciona como infraestrutura de proteção que condiciona o acesso à alimentação adequada, medicamentos, transporte, moradia e cuidados, elementos diretamente associados à prevenção de agravos, ao manejo de doenças crônicas e à redução de vulnerabilidades cumulativas na velhice ^21^.

Do ponto de vista da gestão pública, a persistência da judicialização e a instabilidade do tempo médio de concessão operam como indicadores sensíveis de fricções institucionais que demandam intervenção estrutural: fortalecer critérios e procedimentos administrativos que reconheçam a vulnerabilidade de modo mais consistente e socialmente sensível; investir em integração de bases, padronização de rotinas e cooperação intersetorial com o SUAS; e reduzir a dependência de “portas alternativas” para a efetivação do direito ^26^ ^,^ ^27^.

A questão decisiva não é apenas “se” o benefício existe, mas “como” ele é implementado: se assegura acesso oportuno para quem mais precisa ou se reproduz seletividades que transformam um direito assistencial em um percurso desigual de obtenção de proteção ^19^.

## Conclusão

O BPC permanece como uma política essencial de proteção de renda na velhice e cumpre uma função relevante de segurança econômica para pessoas idosas vulneráveis, assegurando um patamar mínimo de renda a quem não acessa a proteção previdenciária contributiva. Contudo, a efetivação do direito e a capacidade do programa de promover equidade social dependem das condições institucionais de implementação, examinadas aqui com base em três dimensões interligadas: perfil dos beneficiários, vias de concessão e desempenho operacional.

A concentração de beneficiários nas faixas etárias mais jovens da velhice e a predominância feminina, interpretadas à luz de diferenciais de expectativa de vida por sexo e de desigualdades de gênero acumuladas ao longo do curso da vida, evidenciam que o BPC atende de forma mais intensa grupos estruturalmente mais vulneráveis. A hipótese de que as idades mais avançadas enfrentam barreiras adicionais de acesso merece investigação mais aprofundada por análises de cobertura proporcional que desagreguem o efeito demográfico dos possíveis obstáculos institucionais.

A persistência e o crescimento da judicialização indicam fricções que não são resolvidas com ajustes marginais e que podem tornar a efetivação do direito assimetricamente mais provável para quem tem maior capacidade de mobilização jurídica. A instabilidade do tempo médio de concessão, em dias, reforça que a capacidade estatal não é detalhe gerencial, mas condição de efetividade do direito: o atraso reconfigura o benefício em proteção tardia, com custos sociais e sanitários que tendem a ser invisibilizados nos indicadores estritamente administrativos.

Reforçar a governança, a capacidade administrativa e as estratégias de redução de barreiras são condições centrais para ampliar a efetividade e a equidade do BPC como componente da proteção social e dos determinantes sociais da saúde. Este estudo apresenta limitações inerentes ao uso de dados secundários públicos e à ausência de coleta de dados primários, o que impede captar experiências individuais de acesso, trajetórias burocráticas e desistências. Análises de cobertura proporcional e técnicas de regressão segmentada são recomendadas para pesquisas futuras que busquem aprofundar a compreensão dos padrões aqui identificados.

## Data Availability

As fontes de informação utilizadas no estudo estão indicadas no corpo do artigo.

## References

[1] World Health Organization (2025). World report on ageing and health.

[2] Instituto Brasileiro de Geografia e Estatística (2024). Projeção da população do Brasil e das Unidades da Federação.

[3] Brasil (1988). Constituição da República Federativa do Brasil de 1988.

[4] Brasil (1993). Lei nº 8.742, de 7 de dezembro de 1993. Dispõe sobre a organização da Assistência Social e dá outras providências.. Diário Oficial da União.

[5] Duarte CMR, Manzini-Covre MF, Fonseca F, Oliveira SC (2017). Proteção social e política pública para populações vulneráveis uma avaliação do Benefício de Prestação Continuada da Assistência Social - BPC no Brasil. Ciênc Saúde Colet.

[6] Sen A (2000). Desenvolvimento como liberdade.

[7] Rawls J (2003). Justiça como equidade: uma reformulação.

[8] Stopa R (2019). O direito constitucional ao Benefício de Prestação Continuada (BPC) o penoso caminho para o acesso. Serv Soc Soc.

[9] Peres VAP (2024). A desconfiguração do Benefício de Prestação Continuada (BPC) sob o ultra neoliberalismo. Argumentum.

[10] Zaragoza D (2023). O benefício de prestação continuada e o ciclo da política pública judicializada.

[11] Boschetti I (2006). Seguridade social e trabalho: paradoxo na construção das políticas de previdência e assistência social no Brasil.

[12] Ministério do Desenvolvimento e Assistência Social, Família e Combate à Fome Monitora MDS..

[13] Minayo MC (2014). O desafio do conhecimento: pesquisa qualitativa em saúde..

[14] Souza MT, Silva MD, Carvalho R (2010). Revisão integrativa o que é e como fazer. Einstein (São Paulo).

[15] Ministério do Desenvolvimento e Assistência Social, Família e Combate à Fome Secretaria de Avaliação, Gestão da Informação e Cadastro Único..

[16] Instituto Brasileiro de Geografia e Estatística (2025). Pesquisa Nacional por Amostra de Domicílios Contínua (PNADC) 2024.

[17] Ministério do Desenvolvimento e Assistência Social, Família e Combate à Fome VIS DATA 3..

[18] Brasil (2007). Decreto nº 6.214, de 26 de setembro de 2007. Regulamenta o Benefício de Prestação Continuada da assistência social devido à pessoa com deficiência e ao idoso de que trata a Lei nº 8.742, de 7 de dezembro de 1993, e a Lei nº 10.741, de 1º de outubro de 2003 , acresce parágrafo ao art. 162 do Decreto nº 3.048, de 6 de maio de 1999, e dá outras providências.. Diário Oficial da União.

[19] Paiva AB, Pinheiro MB (2021). Proteção social não contributiva e implementação do BPC.

[20] Mesquita ACS (2024). A continuidade institucional do Benefício de Prestação Continuada (BPC) como instrumento da seguridade social..

[21] Instituto de Pesquisa Econômica Aplicada Importância da Previdência Social: beneficiômetro..

[22] Alcântara IT, Mountian AG (2021). Gênero e previdência contexto e evolução das regras previdenciárias para as mulheres brasileiras. Boletim de Políticas Públicas.

[23] Sidone OJG, Giambiagi F, Tinoco G (2024). Política previdenciária e equidade de gênero: propostas para uma maior efetividade..

[24] Becker KL (2020). Distribuição regional e características socioeconômicas do público-alvo do Benefício de Prestação Continuada (BPC). Bol Reg Urb Ambient.

[25] Souza PHGF, Hecksher MD (2025). Pobreza e desigualdade no Brasil no curto e no longo prazo.

[26] Sposati A, Meira PT (2023). Transferência de renda no Brasil: entre a herança recebida e a direção prometida.

[27] Oliveira TF, Rodrigues VER (2019). Anais do III Congresso Internacional de Política Social e Serviço Social.

